# Lack of concordance between residual viremia and viral variants driving de novo infection of CD4^+^ T cells on ART

**DOI:** 10.1186/s12977-016-0282-9

**Published:** 2016-08-02

**Authors:** Maria C. Puertas, Marc Noguera-Julian, Marta Massanella, Christian Pou, Maria J. Buzon, Bonaventura Clotet, Mario Stevenson, Roger Paredes, Julià Blanco, Javier Martinez-Picado

**Affiliations:** 1 AIDS Research Institute IrsiCaixa, Institut d’Investigació en Ciències de la Salut Germans Trias i Pujol, Universitat Autònoma de Barcelona, Badalona, Spain; 2 “Lluita Contra la Sida” Foundation, Hospital Universitari Germans Trias i Pujol, Universitat Autònoma de Barcelona, Badalona, Spain; 3 Universitat de Vic – Universitat Central de Catalunya (UVic-UCC), Vic, Spain; 4 Division of Infectious Diseases, Department of Medicine, University of Miami Miller School of Medicine, Miami, FL USA; 5 Catalan Institution for Research and Advanced Studies (ICREA), Barcelona, Spain; 6 Département de Microbiologie, Infectiologie et Immunologie, Centre de Recherche du CHUM et Université de Montréal, Montreal, Canada; 7 Infectious Diseases Department, Hospital Universitari Vall d’Hebron, Barcelona, Spain; 8 Department of Cell and Molecular Biology, Karolinska Institutet, Solna, Stockholm Sweden

**Keywords:** HIV-1, Viral reservoir, Residual viremia, Persistence, CD4^+^ T cell subsets

## Abstract

**Background:**

In most patients, current antiretroviral therapy (ART) regimens can rapidly reduce plasma viral load. However, even after years of effective treatment, a significant proportion of patients show residual plasma viremia below the clinical detection limit. Although residual viremia might be associated with increased chronic immune activation and morbidity, its origin and its potential role in the replenishment of the viral reservoir during suppressive ART is not completely understood. We performed an in-depth genetic analysis of the total and episomal cell-associated viral DNA (vDNA) repertoire in purified CD4^+^ T cell subsets of three HIV-infected individuals, and used phylogenetic analysis to explore its relationship with plasma viruses.

**Results:**

The predominant proviral reservoir was established in naïve or memory (central and transitional) CD4^+^ T cell subsets in patients harboring X4- or R5-tropic viruses, respectively. Regardless of the viral tropism, most plasma viruses detected under suppressive ART resembled the proviral reservoir identified in effector and transitional memory CD4^+^ T-cell subsets in blood, suggesting that residual viremia originates from these cells in either blood or lymphoid tissue. Most importantly, sequences in episomal vDNA in CD4^+^ T-cells were not well represented in residual viremia.

**Conclusions:**

Viral tropism determines the differential distribution of viral reservoir among CD4^+^ T-cell subsets. In spite of viral tropism, the effector and transitional memory CD4^+^ T-cells subsets are the main source of residual viremia during suppressive ART, even though their contribution to the total proviral pool is small. However, the lack of concordance between residual viremia and viral variants driving de novo infection of CD4^+^ T cells on ART may reflect the predominance of defective plasma HIV RNA genomes. These findings highlight the need for monitoring the multiple viral RNA/DNA persistence markers, based on their differential contribution to viral persistence.

**Electronic supplementary material:**

The online version of this article (doi:10.1186/s12977-016-0282-9) contains supplementary material, which is available to authorized users.

## Background

Current antiretroviral therapy (ART) can control viremia in a few weeks, and its extensive use has notably decreased mortality and morbidity rates among individuals infected by human immunodeficiency virus type 1 (HIV-1). However, complete clearance of the infection is never achieved, and plasma viremia rebounds, with very few exceptions, if treatment is discontinued [1]. The persistence of HIV-1 is believed to be a consequence of a population of latent proviruses that are established early during the primary infection and remain dormant for years, mostly in long-lived memory CD4^+^ T cells [2–7].

In the last decade, the use of ultrasensitive technologies to measure viral load has made it possible to detect residual viremia (HIV-1 RNA levels below 50 copies/mL), even after many years of effective ART [8–10]. As free HIV-1 virions have a short circulating half-life, residual viremia evidences recent virus production by an “active” reservoir during effective ART. The potential consequences of this constant supply of viral antigens for chronic immune activation are not entirely clear [11, 12]. On the other hand, antiretroviral treatment intensification does not lower the levels of residual viremia [13–19], and data from phylogenetic analyses of the viruses found in the plasma of ART-treated individuals show a lack of long-term genetic evolution [20–23] suggesting that residual viremia does not largely reflect ongoing viral replication. Still, it is not fully established whether the source of this active viral production is a particular cell type or anatomical compartment, in which antiretroviral treatment might be preventing new infection events [24, 25]. Indeed, residual viremia might also be the result of small bursts of viral production derived from clone-specific T-cell activation [26–29] or a combination of both mechanisms. This issue is of particular interest, as the therapeutic approaches to be considered when trying to reduce the chronic immune activation that is potentially derived from residual viremia will depend on the origin and specific target cell populations.

In contrast to the long-term stability of integrated proviral genomes, episomal vDNA is considered a more dynamic, surrogate marker of recent infection events [30–34]. Thus the detection of episomal vDNA molecules in the peripheral blood mononuclear cells (PBMCs) of some patients on ART or after treatment intensification with an integrase inhibitor, suggests some degree of de novo infection may persist in cellular or anatomical reservoirs that may be partially refractory to antiretroviral drugs [13, 35, 36]. The actual origins of the virus that fuels the infection events revealed by episomal sequences are unknown as is the role of residual viremia in this “cryptic” viral replication.

In order to identify the source of residual viremia during ART, we isolated different T-cell subsets from peripheral blood and genetically characterized their proviral repertoire using ultra-deep sequencing. We also analyzed episomal vDNA to characterize the viral populations driving de novo infections in this scenario. Our results indicate that: (1) viral sequences in residual viremia are predominantly related to proviral sequences in effector and transitional memory CD4^+^ T-cells suggesting that residual viremia originates from these cells, and (2) there is limited sequence relationship between episomal vDNA and plasma viral RNA, suggesting that viruses in plasma are not the source of the de novo infection events detected in peripheral CD4^+^ T-cells.

## Results

### Patient characteristics and treatment outcome

**Fig. 1:**
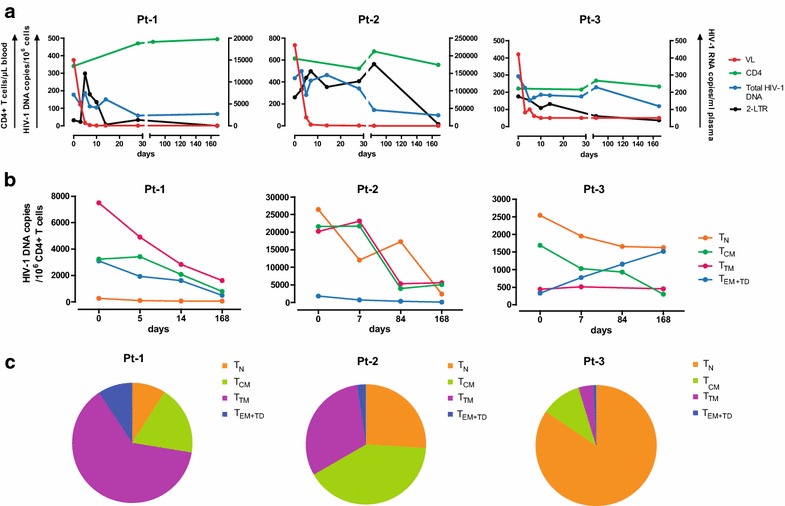
Treatment outcome and infection dynamics in CD4^+^ T-cell subsets. **a** CD4^+^ T-cell counts and viral dynamics, including plasma viral load, total vDNA content, and 2-LTR episomes in PBMCs, were measured up to 6 months after switching treatment in each patient. **b** Cell-associated vDNA content was also measured by qPCR in each purified CD4^+^ T-cell subset at baseline, at week 1 or 2, and also after viral suppression was achieved. **c** Relative contribution of each CD4^+^ T-cell subset to the total pool of infected cells in each patient was calculated according to the vDNA content and the frequency of each subset in the whole CD4^+^ T-cell population at baseline

A previous clinical trial performed in our hospital (Ithaca; NCT00685191) included 15 antiretroviral-experienced HIV-infected patients who switched to a raltegravir-based salvage regimen at study entry. For further ultra-deep sequencing analysis, we selected the five patients with the highest levels of total and episomal cell-associated vDNA in PBMCs; consistent proviral HIV-1 *Env* amplification in the different subsets was obtained from 3 individuals at baseline and after viral suppression (Table 1; Fig. 1a).

**Table 1:** Patient characteristics at baseline

	Pt-1	Pt-2	Pt-3
Age	44	61	43
Gender	Female	Male	Male
Years from HIV-1 diagnosis	11.3	12.5	17.4
Number of previous ART regimens	7	9	5
Nadir CD4^+^ T-cells (cells/μL)	139	145	7
Plasma Viral Load (HIV RNA copies/mL)	15,000	230,000	421
CD4^+^ T cells (cells/μl)	341	614	221
Total HIV-1 DNA in PBMC (cp/10^6^ cells)	177.7	434.8	292.7
2-LTR circles in PBMC (cp/10^6^ cells)	31.8	260.5	175.4
Salvage regimen	RAL, ETV, DRV/r	RAL, 3TC, DDI, DRV/r	RAL, TDF, FTC, DRV/r

### Contribution of the different CD4^+^ T-cell subsets to the establishment of viral reservoirs

We characterized four CD4^+^ T-cell subsets according to the differential expression of the surface markers CD45RA, CCR7, and CD27, as follows: naïve (T_N_: CD45RA^+^CCR7^+^CD27^+^), central memory (T_CM_: CD45RA^−^CCR7^+^CD27^+^), transitional memory (T_TM_: CD45RA^−^CCR7^−^CD27^+^), and effector memory plus terminally differentiated cells (T_EM+TD_: CD45RA^+/−^CCR7^−^CD27^−^) (Additional file 1: Fig. S1). After purification of each subpopulation by fluorescence-activated cell sorting (FACS) and quantification of HIV-1 DNA by qPCR, we observed a generalized reduction in the vDNA content in all patients and in all four subsets upon initiation of rescue therapy (Fig. 1b). However, the proportion of each subset in peripheral blood and the relative contribution of each subset to the total pool of infected cells were notably different between the patients but quite consistent over time despite viral suppression (Fig. 1c, and Additional file 2: Fig. S2). In Patient 1 (Pt-1) the T_TM_ subset was preferentially infected (>50 % of the total pool of infected CD4^+^ T cells), followed by the T_CM_ subpopulation (19 %). In Patient 2 (Pt-2) the T_N_, T_CM_, and T_TM_ subsets were extensively infected, and their contribution to the total pool of infected cells was equivalent. In Patient 3 (Pt-3), however, the memory subsets (T_CM_ and T_TM_) bore only a small proportion of infected CD4^+^ T cells, and the T_N_ subpopulation was the main target of viral infection (>80 % at all the time points analyzed). The only common feature in all subjects was the relatively low contribution of the T_EM+TD_ subsets to the total pool of infected cells (<10 % in all patients and at all the time points analyzed), which was due to the small number of these cells found in peripheral blood and/or their low infection frequency (Additional file 2: Fig. S2).

### Distribution of proviral reservoir among CD4^+^ T-cell subsets is determined by viral tropism

**Fig. 2:**
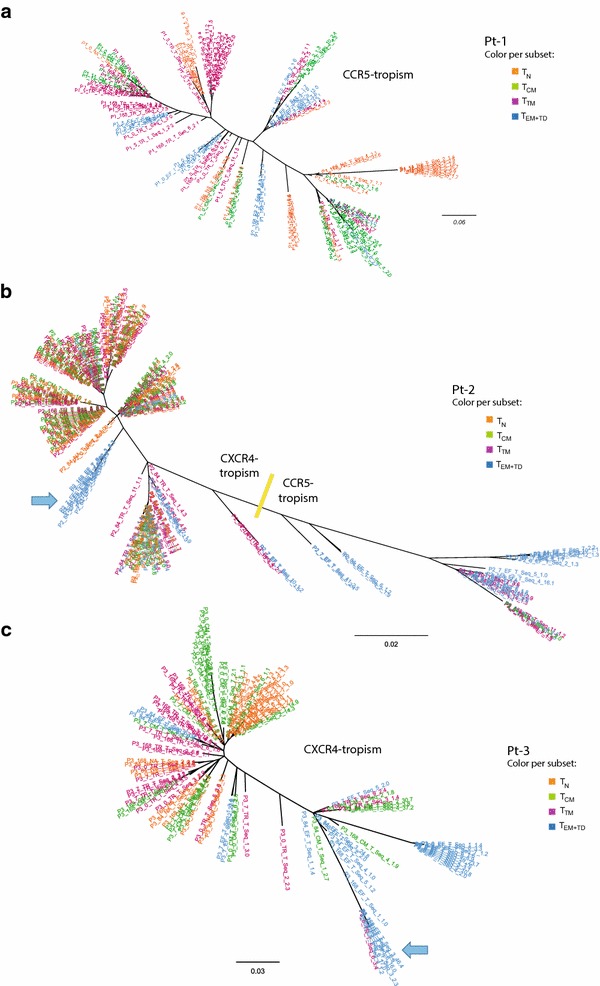
Phylogenetic analysis of the whole vDNA integrant pool. Maximum likelihood phylogenetic trees (unrooted) showing the cell-associated vDNA repertoire harbored by the different CD4^+^ T-cell subsets (color coded). Viral sequences from the four time points analyzed (pre- and post-treatment switching) are included in the analysis. The overall result from the Env-tropism prediction is indicated for each tree: **a** Patient 1; **b** Patient 2; **c** Patient 3. Particular branches, composed mainly by T_EM+TD_ proviral sequences and detected at different time points, are indicated by *blue arrows*

The HIV-1 Env-V3 region was amplified from the total DNA fraction of each purified CD4^+^ T-cell subset and analyzed using ultra-deep sequencing. In order to provide a general overview of the proviral repertoire, we first constructed phylogenetic trees with proviral sequences from samples taken at baseline (plasma viral load >200 RNA copies/mL) and at three additional time points from week 1 and up to week 24, after treatment switch. In general, proviral sequences from the different cellular subpopulations intermingled in the three subjects (Fig. 2). Similarly, the sequence space had minor chronological compartmentalization (Additional file 3: Fig. S3). Interestingly, in Pt-2 and Pt-3 we observed some distinguishable clusters composed mainly—if not exclusively—of T_EM+TD_ cells, occasionally including proviral sequences from multiple time points (indicated by blue arrows in Fig. 2 and S3).

The viral tropism, as predicted by the Geno2Pheno algorithm, revealed profound differences between the three study subjects: all viral sequences from Pt-1 were CCR5-tropic, those from Pt-3 were CXCR4-tropic, and there was a mixture between CCR5- and CXCR4-tropic sequences in Pt-2. Indeed, in the phylogenetic tree from Pt-2, CXCR4-tropic quasispecies (indicated in Fig. 2b) included most of the sequences from T_N_, T_CM_, and T_TM_ CD4^+^ T cells, while CCR5-tropic proviruses belonged mainly to the effector subsets: the proportion of CCR5-tropic Env variants found in T_EM+TD_ cells ranged from 33 to 61 % at the different time points analyzed, although they represented less than 15 % in the other cell subsets (data not shown). Overall, these differences in viral tropism may explain the differential contribution of the T_N_ CD4^+^ T-cell subset to the total pool of infected cells in each subject (Fig. 1c), as their susceptibility to HIV-1 infection is highly dependent on CXCR4 co-receptor usage.

### Long-lived persistence of archival proviruses in highly-differentiated CD4^+^ T-cells

**Fig. 3:**
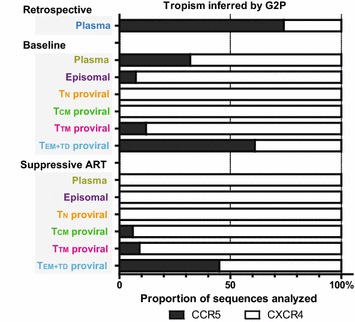
Dynamics of the CCR5/CXCR4 tropism proportion in the cell-associated vDNA in Patient 2. The proportion of X4/R5-tropic sequences, as inferred by Geno2Pheno algorithm, is indicated for each sample. Results from the retrospective plasma sample are shown, together with samples from baseline and a time point after viral suppression. Likewise, data from the contemporaneous proviral DNA sequences from the purified CD4^+^ T-cell subsets, and the episomal vDNA molecules from total PBMCs, are shown for comparison

Because of differences in levels of co-receptor expression and intrinsic cellular half-life, the mixed viral tropism found in Pt-2 offers a unique possibility to further evaluate and compare the contribution of each CD4^+^ T-cell subset to maintenance of the HIV-1 reservoir. For this purpose, viral evolution was further assessed by additional analysis of a retrospective plasma sample, collected 11 years before the baseline of the present study. At the time of retrospective sampling, the patient was receiving antiretroviral therapy and had a plasma viral load of 18,000 vRNA copies/mL. As shown in Additional file 4 (Fig. S4), viral sequences detected in the retrospective sample were mostly distributed at the CCR5-tropic branches of the phylogenetic tree, with only some variants located in a cluster that could have represented intermediate variants in the transition to CXCR4 tropism. In contrast, at baseline of the present study, the patient’s viruses showed a predominance for CXCR4-tropic variants at both the plasma and the proviral level (Fig. 3), with no signs of intermediate variants, which were only detected again as proviruses after switching treatment (Fig. S3). In fact, CCR5-tropic and transitional variants were mostly found as proviruses in the T_EM+TD_ subset (Figs. 2b, 3), although T_CM_ and T_TM_ are also susceptible to infection by CCR5-tropic viruses. The high prevalence of CCR5-tropic sequences in this subset in the absence of reservoir replenishment might reflect the long-term survival of highly differentiated CD4^+^ T-cell clones or the differentiation of other long-lived memory cells bearing archival proviruses.

### Divergence between residual viremia and de novo infection events under effective ART

**Fig. 4:**
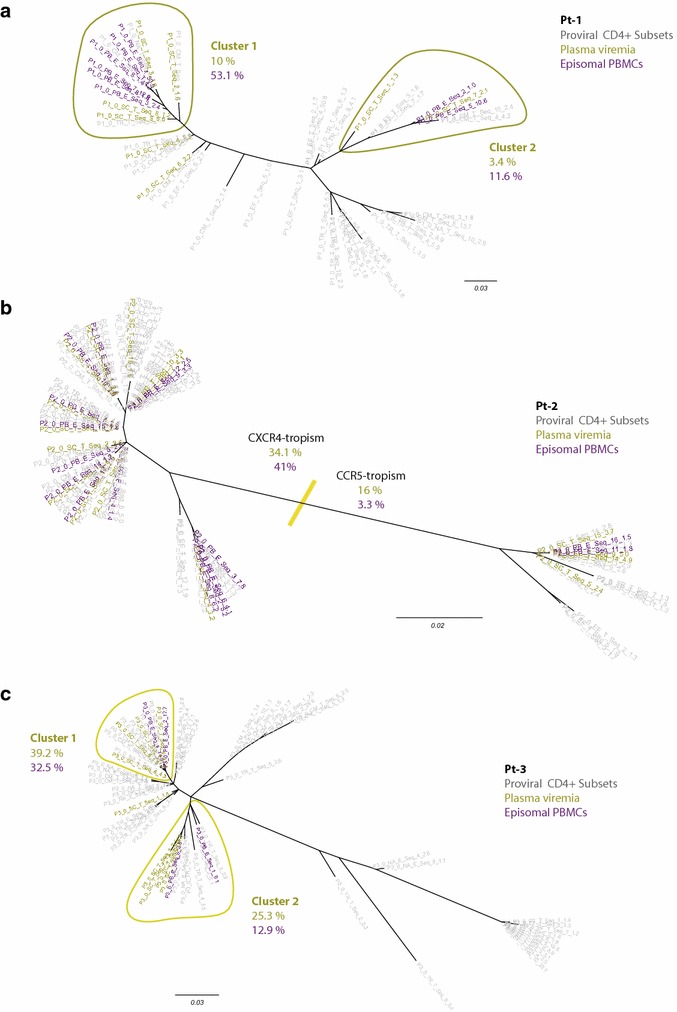
Viral diversity during active replication. Maximum likelihood phylogenetic trees (unrooted) of viral variants detected at virologic failure (just before treatment switch): **a** Patient 1; **b** Patient 2; **c** Patient 3. Plasma viremia sequences (*gold*) and episomal vDNA from PBMCS (*violet*) are highlighted; proviral DNA from the CD4^+^ T-cells (*grey*) is also included. Major plasma/episomal clusters are highlighted when clearly distinguished (Pt-1 and Pt-3), and the frequency of sequences included is color-coded at each tree (in percentages). In B (Pt-2), the overall result from the Env-tropism prediction is indicated

At baseline, we performed a phylogenetic analysis for each subject including viral sequences from plasma vRNA, episomal vDNA (from total PBMCs), and total cell-associated vDNA from each CD4^+^ T-cell subset. In all subjects, we observed a high degree of similarity between major episomal and plasma viral clusters (Fig. 4). Proviral DNA sequences from all CD4^+^ T-cell subsets in the absence of therapy were found intermingled and reflecting mostly the actively replicating virus population.

**Fig. 5:**
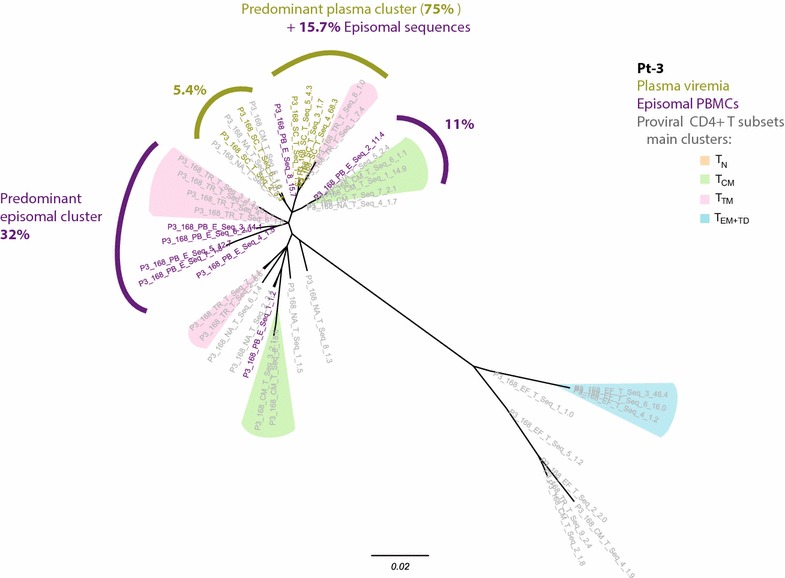
Analysis of residual viremia during effective ART in Pt-3. Maximum likelihood phylogenetic tree (unrooted) of the viral quasispecies detected 24 weeks after switching treatment, showing plasma viremia sequences (*gold*), episomal vDNA from PBMCS (*violet*), and proviral DNA sequences from the CD4^+^ T-cells (*grey*). Predominant plasma clusters and episomal clusters are indicated, and the total amount of sequences included (in percentages) is also indicated. *Color shading* identify branches containing >5 % of the proviral sequences from each subset. Sequences from T_N_ cells were specially dispersed along the tree, so no specific clusters are indicated

Next, to evaluate the nature of residual viremia and cryptic viral replication under effective ART we performed the phylogenetic analysis of those samples taken when the patients had achieved viral suppression. Despite this analysis was not performed after long-term suppression to avoid resampling bias, we selected a sampling time frame contained in the third-phase decay kinetics of plasma HIV-RNA after Raltegravir-based treatment initiation, in which most plasma viruses are presumed to come from latently infected cells that become activated [37]. The specific objectives of these analyses were as follows: (1) to identify whether a specific peripheral CD4^+^ T-cell subset is the origin of residual viremia under suppressive ART, and (2) to determine whether plasma virions fuel de novo infection events–as revealed by episomal cDNA species–in peripheral CD4^+^ T-cells from individuals on suppressive ART. For that purpose, we only used those samples from which the plasma HIV-1 vRNA and the episomal vDNA from PBMCs had been successfully amplified and sequenced. In the case of Pt-3 (Fig. 5), we observed a major plasma cluster including a highly predominant plasma clone and a portion (15.7 %) of the episomal sequences. However, the main episomal cluster (32 % of the episomal sequences) contained no plasma sequences. Therefore, although plasma and episomal viral quasispecies were partially intermingled, most recent infection events evidenced by specific episomal vDNA were not closely related to the predominant plasma clone under effective ART. Only proviral sequences from the T_TM_ subset were included in the predominant clusters of both active viral compartments, as active virion-producing cells, target cells, or both.

### Effector and transitional memory CD4^+^ T-cell subsets are the main active reservoirs

**Fig. 6:**
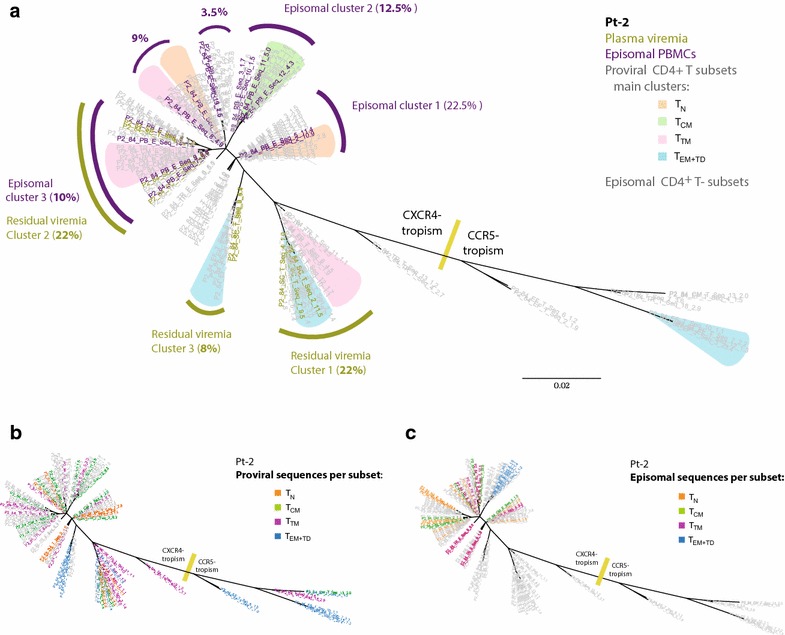
Analysis of residual plasma viruses on effective ART in Pt-2. Maximum likelihood phylogenetic tree (unrooted) of the plasma, proviral, and episomal viral variants detected 12 weeks after switching treatment. **a** Plasma viremia sequences (*gold*) and episomal vDNA from PBMCs (*violet*) are highlighted. Predominant plasma clusters and episomal clusters are identified, and the proportion of sequences included (in percentages) are indicated. In the same tree, *color shading* identify branches containing >10 % of the proviral sequences from each subset. The overall distribution of proviral versus episomal sequences are shown in (**b)** and (**c**), respectively, color-coded according to the CD4^+^ T-cell subset they come from. In all trees, the overall result from the Env-tropism prediction is indicated

In Pt-2, no predominant plasma clone was detected after treatment switching (Fig. 6a). Instead, we identified three CXCR4-tropic clusters, two of which contained 22 % each and one included 8 % of all sequences obtained from the plasma sample. Most sequences co-localizing in these clusters matched with proviral sequences that were particularly prevalent in T_EM+TD_ and T_TM_, thus indicating their major role in residual viremia production, either in blood or in cell-equilibrated lymphoid tissue. Most episomal sequences from PBMCs were not well represented in these viremia-containing clusters, again suggesting that much residual viremia does not derive from, nor contribute to, productive replication in peripheral blood.

In Pt-2, episomal vDNA from the four purified CD4^+^ T-cell subsets was successfully sequenced and included in the phylogenetic tree, so that the differential distribution of proviral and episomal viral variants harbored by each CD4^+^ T-cell subset was examined (Fig. 6b, c). The segregation of related proviral and episomal viral sequences at different CD4^+^ T-cell subsets, as observed in episomal clusters 2 and 3, indicates the occurrence of cross-infection events between them.

## Discussion

**Fig. 7:**
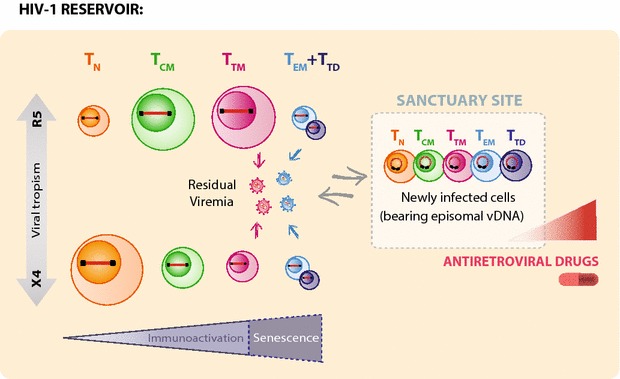
Proposed model for the composition of HIV-1 reservoirs on ART. The relative contribution of each CD4^+^ T-cell subset to the total pool of infected cells during ART greatly depends on the history of viral tropism of the patient, as X4-tropism significantly increases the long-term reservoir in naïve CD4^+^ T cells. Regardless of the viral tropism, T_TM_ and T_EM+TD_ cells seem to be the main producers of residual viremia, despite the relative proportion of infected T_EM+TD_ is invariably small. However, de novo infection of CD4^+^ T cells under suppressive ART is driven by viral populations poorly represented in residual viremia. Instead, cryptic viral replication presumably takes place in anatomical sanctuary sites (presumably at lymphoid tissue), where clonal activation, cell-to-cell transmission and suboptimal antiretroviral drug concentration might enhance the chances of new infection in all CD4^+^ T-cell subsets. Newly infected cells, identified because of their particularity of bearing episomal vDNA can then migrate to periphery and be detected in blood samples. It remains to be determined if plasma viremia discordance is determined by anatomical compartmentalization of productively infected cells (plasma virions may be produced by circulating CD4^+^ T cells or in a different anatomic location that is less susceptible to ART) or to an indirect effect of differential replicative capacity of proviral variants

HIV-1 preferentially infects activated CD4^+^ T cells, although resting CD4^+^ T cells may also be infected, albeit to a lesser extent [38–40]. In most cases, productive infection results in the rapid death of infected cells, but a small proportion of these cells can revert to a long-lived resting phenotype and establish persistent viral reservoirs [41]. Consequently, the susceptibility of CD4^+^ T-cell subpopulations to HIV-1 infection, in addition to their mean half-life and homeostatic proliferation, is a key factor in the contribution of each subset to viral persistence in long-term virologically suppressed patients [42–47]. In this study, we evaluated the relative contribution of different CD4^+^ T-cell subsets to the total pool of infected cells, both in virologic failure and after effective treatment switching. Despite the limited number of patients included in the study, we observed high heterogeneity between them in the distribution of the subsets in the viral reservoir. In line with most reported cases, we found that most of the proviral DNA remained in T_TM_ and T_CM_ CD4^+^ T cells in the patient harboring a pure CCR5-tropic virus [45]. However, our results also evidence the long-term stability of viral reservoirs in naïve CD4^+^ T cells when the infection is driven by CXCR4-tropic viruses, as is the case of Pt-3, in whom T_N_ cells account for >80 % of the total pool of infected cells at all the time points evaluated. An interesting intermediate situation was observed in the patient harboring a mixed X4/R5 viral population. These data are in accordance with previous studies showing higher susceptibility of naïve CD4^+^ T cells to the X4-mediated infection and preferential detection of X4 proviral variants in this subset during suppressing ART [48–52]. We cannot rule out the possibility that a small portion of these cells correspond to the T_SCM_ phenotype, despite they have been described to be more susceptible to R5-tropic HIV-1 [46, 53]. Our results highlight the key role of long-lived T_N_ CD4^+^ T cells as a potential target for future therapeutic interventions aimed at the reactivation and/or specific targeting of the latent reservoir in patients in whom X4-tropic viruses may be detected (Fig. 7).

In general, the repertoire of proviral sequences found at the different CD4^+^ T-cell subsets showed a mixed genetic population in all patients, possibly indicating cross-infection events between subsets and/or migration events of proviral quasispecies as a result of cellular differentiation from one functional phenotype to another, as previously described for resting versus activated CD4^+^ T-cell subsets [54, 55].

The diversity of the tropism found in viral quasispecies from Pt-2 enabled us to elucidate the direction of viral evolution throughout the course of the infection. It also offered us the possibility of evaluating the contribution of each CD4^+^ T-cell subset to the long-lived reservoir. In this sense, although all memory CD4^+^ T cells are equally prone to infection by CCR5-tropic viruses, the proportion of proviruses carrying archival CCR5-using Env variants was clearly higher in the T_EM+TD_ subsets at all the time points analyzed, despite the predominance of CXCR4-tropic viruses in the plasma virus population during virologic failure. Transitional intermediate variants might even be represented by minor clusters detected in the effector and transitional memory compartments and located at the CCR5- to CXCR4-tropism transition area of the phylogenetic tree. Presumably, the low fitness of these viral variants would account for their low frequency, as they are only detected as proviruses when viral replication is inhibited by antiretroviral therapy [56]. However, further functional assays would be needed to confirm this hypothesis.

The fact that some T_EM+TD_ proviral clusters were recurrently detected at different time points after treatment change reinforces the hypothesis that the highly-differentiated effector CD4^+^ T-cell populations may be predominant reservoirs of archival proviruses, probably by means of long-term persistence of clonally expanded populations during ART, as other authors have also shown [47, 57]. Albeit our sorting strategy impeded segregation of T_EM_ and T_TD_ cells, the extremely low frequency of the latter in peripheral blood [30] might lead to speculate that most of the cell-associated vDNA detected in this subset lies in T_EM_ cells [45, 58]. T_EM_ are short-lived in nature, and this requirement for continuous replenishment in vivo has been shown to be compensated with a rapid proliferation rate [30]. This intrinsic feature of T_EM_ CD4^+^ T cells increases the likelihood of this particular subset to harbor clonally expanded proviral variants. Further studies might elucidate whether the long-term survival of these stable populations is induced by homeostatic proliferation, chronic antigen stimulation or vDNA integration in host genes involved in regulation of cell proliferation [45, 47, 59–62] and if these cells can eventually become a source of viral production.

Residual viremia in patients on ART has been extensively reported, but its contribution to chronic immune activation is not entirely clear. Moreover, the replication potential of residual plasma viruses and whether they are able to prime viral rebound upon treatment interruption remain uncertain [63–65]. Indeed, the identification of the cellular source of residual viremia has been an active research field in recent years, and the data published to date showed genetic discordances with major vDNA populations in the PBMCs of patients on suppressive ART and even in partially effective therapy [21, 28, 55, 66–68]. Some reports had investigated the feasibility of the CD4^+^ T-cell population itself and the circulating monocytes, as being responsible for residual viral production in treated patients; however, poor genetic identity between viral RNA and proviruses was reported for both cell types [63, 69]. These observations led us to question whether the cellular source of residual viremia was indeed a minor population in PBMCs or, alternatively, a tissue-restricted cell type. In this regard, recent studies have shown a poor genetic relationship between plasma viremia and tissue-specific reservoirs such as gut-associated lymphoid tissue and cerebrospinal fluid [47, 70]. Thus, we performed an in-depth analysis of the predominant plasma viral variants during antiretroviral treatment and compared them with the vDNA repertoire from different CD4^+^ T-cell subpopulations isolated by phenotype-based cell sorting. We found that residual viremia sequences were preferentially clustered with proviral variants prevailing in the T_EM+TD_ and T_TM_ CD4^+^ T-cell subsets. Of note, the lack of matching episomal vDNA sequences suggests that T_EM+TD_ and T_TM_ CD4^+^ T-cell subsets are most likely the source of residual viremia. This might be explained in part by the fact that T_TM_ and T_EM_ CD4^+^ T-cell subsets typically present higher activation rates [71], thus providing a suitable scenario for viral production. It is interesting that in both individuals analyzed, the vDNA sequences most closely related to residual viremia are found in CD4^+^ T-cell subsets that represent a significantly small portion of their total proviral reservoir, as inferred from the data in Fig. 1. This might also be a reason for the lack of genetic similarity in previous experimental approaches, in which these CD4^+^ T-cell subsets were not specifically sorted prior to vDNA characterization. Future studies would verify if these cell subsets are indeed transcriptionally active during viral suppression and its potential role in viral rebound after treatment interruption [64].

In this study, we also aimed to evaluate the potential relationship between residual viremia and potential de novo infection under suppressive ART. Our first comparative analysis, which was based on samples collected at the time of virological failure, clearly illustrated the linkage between viral populations present in plasma and episomal vDNA, as expected on active viral replication [72, 73]. Interestingly, a different scenario was observed when we analyzed subsequent samples on suppressive therapy. Antiretroviral suppression led to minor co-localization between major plasma clusters and episomal viral quasispecies, suggesting that, in this setting, most of the viruses detected in plasma are not responsible for de novo infection events. To our knowledge, these are the first studies comparing the genetic composition of these two viral populations. The dynamic repertoire observed (major clusters are not coincident at baseline and after treatment change) suggests the short-lived nature of both plasma and episomal viral populations, in contrast to the stable nature of proviral reservoir [74]. The significant discordance between residual viremia and recent infection events might be due to anatomical compartmentalization of those two “active” reservoirs, and supports the potential existence of anatomical reservoirs in which optimal intracellular drug levels might not be achieved, thus favoring local viral replication (illustrated in Fig. 7). Such would be the case of lymphoid tissues, where the concentrations of some antiretroviral drugs are lower than in peripheral blood and where close contact between T cells might enhance cell-to-cell viral transmission [75]. In this context, clonal cell activation of latently infected memory CD4^+^ T cells might lead to temporary and locally limited bursts of viral reinfection in proximally activated target cells. Such events might lead to spatial compartmentalization of infected foci in lymphoid tissue, as previously described [76, 77], thus replenishing viral reservoir despite not driving systemic linear viral evolution. Subsequent mobilization from lymphoid tissues to blood might then enable detection of recently infected cells in peripheral blood [78]. Actually, a limitation of the present study is that lymphoid tissue samples were not available from these patients, so we were not able to confirm this hypothesis. Likewise, further characterization of these infection foci in this compartment would be of major interest for the HIV cure prospects, as it has been reported that a significant number of rebounder/founder variants emerge from multifocal infection in lymphatic tissues after treatment interruption [79]. In addition, rapid virion clearance by the reticuloedothelial system [80] and deposition of virions on FDCs, may limit de novo infection by virions produced in lymphoid tissue to cells in close anatomic proximity and reduce the likelihood of those virions reaching the periphery.

The lack of concordance between residual viremia and viral variants driving de novo infection of CD4^+^ T cells on ART, might also reflect the relative abundance of functional genomes, these being over-represented in the episomal pool. From the total HIV proviral reservoir, only a fraction of viral genomes are competent for production of new virions, and a small percentage of those—the ones that are revealed by episomal sequencing—will be infectious. This hypothesis does not exclude the possibility that persistent plasma viremia under ART may contain replication-competent viral variants, either coming from transcriptionally active and eventually clonally-expanded CD4^+^ T cells [62, 64, 65, 81]. Thus, residual viremia may also pose a major concern with regard to viral recrudescence whether ART is discontinued.

## Conclusions

Overall, our results led us to gain insights into the nature of latent HIV-1-reservoir (Fig. 7), evidencing that highly-differentiated CD4^+^ T cell clones can constitute a particularly long-lived proviral reservoir and that naïve CD4^+^ T cells can also establish a significant vDNA reservoir in patients harboring X4-tropic viruses. Likewise, we have observed that effector and transitional memory cells are the main active producers of residual viremia in ART-treated patients, despite their relatively small contribution to the total vDNA integrant pool. Most importantly, viruses detected in plasma are not largely responsible for de novo infection events detected in circulating CD4^+^ T cells. This origin discordance, either due to limited infectivity of plasma viruses or to anatomic compartmentalization of productive infection indicates the relevance of monitoring those multiple viral RNA/DNA persistence biomarkers, based on their potential contribution to viral persistence.

## Methods

### Study subjects

The study included ART-experienced HIV-1-infected subjects who initiated a raltegravir-containing salvage ART regimen comprising at least 3 active drugs. Samples were obtained at several time points during the first 15 days after initiation of raltegravir and at months 1, 3, and 6 thereafter. All subjects provided their signed informed consent to participate into the study. The Ethics Committee of “Germans Trias i Pujol” Hospital approved the study on 21 December 2007, reference #: AC-07-107.

### Sorting of cell subsets

Cryopreserved aliquots of PBMCs were quickly thawed and stained with the following antibody combination: CD3-APC-Cy7 (Clone SK7), CD4-PerCP-Cy5.5 (Clone SK3), CD8-V500 (Clone RPA-T8), CD45RA-V450 (Clone HI100), CCR7-PE-Cy7 (Clone 3D12), and CD27-APC (Clone MT-271, all antibodies were from BD Biosciences). The combination was washed and immediately sorted in a FACSAria cell sorter (BD Biosciences). The gating strategy and a representative example of cell sorting is shown in Additional file 1 (Fig. S1). DNA extraction was performed immediately after cell sorting to avoid cell loss.

## HIV-1 DNA quantification

Total DNA was obtained from whole PBMC samples and from the purified subsets (QIAamp DNA Blood Mini Kit, Qiagen). Total vDNA was quantified by real-time PCR using a set of primers and probe located at the 5′LTR region (RU5 Forward: 5′-TTAAGCCTCAATAAAGCTTGCC-3′; RU5 Reverse: 5′-GTTCGGGCGCCACTGCTAG-3′; RU5 Probe: 5′-CCAGAGTCACACAACAGACGGGCA-3′) [82], while episomal 2-LTR molecular forms were quantified using a set of primers and probe flanked the 2-LTR junction (New C1 forward: 5′-CTAACTAGGGAACCCACTGCT-3′; C4R reverse: 5′-GTAGTTCTGCCAATCAGGGAAG-3′; 2nr4nr probe: 5′-AGCCTCAATAAAGCTTGCCTTGAGTGC-3′). CCR5 gene copies were also estimated to calculate the relative number of HIV-1 DNA copies per million cells (CCR5-F: 5′-GCTGTGTTTGCGTCTCTCCCAGGA-3′; CCR5-R: 5′-CTCACAGCCCTGTGCCTCTTCTTC-3′; CCR5 Probe: 5′-AGCAGCGGCAGGACCAGCCCCAAG-3′). In all qPCR experiments, serial dilutions of the 2LTR-CCR5 plasmid were used to plot the standard curve [36].

### Viral RNA

Ultracentifugation of 3 mL of each plasma sample was followed by a manual guanidinium thiocyanate–based RNA extraction protocol. After precipitation with isopropanol, RNA was eluted in RNAse-free water and subsequently reverse-transcribed using HIV-1-specific primers.

### *Env* amplification for deep parallel sequencing analysis

Primers amplifying the V3 and V4 coding regions of the *env* gene (LA11: 5′-CACAGTACAATGTACACATGGA-3′; Env7: 5′-AGGGGCATACATTGCTTTTCCTA-3′) were used in the one-step RT-PCR of the viral RNA obtained from plasma samples (Superscript III and Platinum Taq High Fidelity, Invitrogen) and also in the first outer PCR amplification from the cell-associated DNA samples (Platinum Taq High Fidelity, Invitrogen).

Primers located upstream of *env* and downstream of the 5′LTR region, respectively (EnvA: 5′-TAGAGCCCTGGAAGCATCCAGGAAG-3′; LA17: 5′-TCTCCTTCTAGCCTCCGCTAGTCAA-3′), were used in the first outer PCR to specifically amplify the envelope region of episomal vDNA (containing either 1 or 2 LTRs) [33].

### Deep sequencing protocol

The first-round PCR products described above were used as a template for a nested-PCR (Platinum^®^ Taq High Fidelity, Life Technologies, Paisley, UK) based on the following 454-adapted primers: V3-454F (HXB2 coordinates 7010-7029) and V3-454R (HIV-1_HXB2_ position 7315-7332). The primers included the corresponding A and B 454 adapters, a 10-mer multiple identifier and a TCAG sequence tag at the 5′ end. PCR products were purified using AMPure Magnetic Beads (Beckman Coulter Inc, Brea, California, USA). The concentration and quality of each amplicon was determined by fluorometry (PicoGreen, Life Technologies, Paisley, UK) and spectrophotometry (Lab-on-a-Chip, Agilent Technologies, Foster City, California, USA). Equimolar pools were made to perform emulsion PCR using a 454-FLX sequencing platform with titanium chemistry (454 Life Sciences/Roche).

### Sequencing analysis

Unique collapsed sequences were obtained using AVA software (v 2.7.0) for all samples, as was the representation of their frequency within each sample. Only sequences with a frequency of ≥1 % within the corresponding sample were used for phylogenetic analysis. Multiple sequence alignments were created using MAFFT software [83]. Maximum likelihood phylogenetic analysis was performed using PhyML(v3.1) [84]. The best nucleotide substitution model was selected for each alignment, and a maximum likelihood phylogenetic tree was calculated using the Subtree Pruning and Regrafting (SPR) algorithm and a 100 bootstrap value.

 Tropism prediction for each of the sequences was inferred using the Geno2Pheno algorithm (www.geno2pheno.org) with a 10 % FPR threshold.

## Additional files

10.1186/s12977-016-0282-9 Sorting strategy used to purify the different CD4^+^ T-cell subsets.
10.1186/s12977-016-0282-9 Infection dynamics in CD4^+^ T-cell subsets. A. Frequency of each CD4^+^ T-cell subset at the different time points after switching treatment, as analyzed by phenotype-based flow cytometry. B. Relative contribution of each CD4^+^ T-cell subset to the total viral reservoir according to the vDNA content and the frequency of each subset in the whole CD4^+^ T-cell population at each time point analyzed.
10.1186/s12977-016-0282-9 Temporal stability of the vDNA integrant pool. In the same phylogenetic tree of Figure 2 the sequences from the four time points analyzed (pre- and post- treatment switching) are identified color-coded. Particular branches, composed mainly by T_EM+TD_ proviral sequences and detected at different time points, are indicated by blue arrows and zoomed in.
10.1186/s12977-016-0282-9 Evolution of the co-receptor tropism in Pt-2. Proviral DNA sequences obtained from the four time points have been used to build the maximum likelihood phylogenetic trees used as a backbone. Viral sequences obtained from a retrospective plasma sample are highlighted in blue. The result from the co-receptor tropism prediction is indicated.
