# Supplementary figures and images for: Lack of concordance between residual viremia and viral variants driving de novo infection of CD4+ T cells on ART
Source: Retrovirology. 2016 Aug 2;13:51. doi: 10.1186/s12977-016-0282-9 (PMC4970251; doi:10.1186/s12977-016-0282-9)

# Additional file 1: Figure S1.

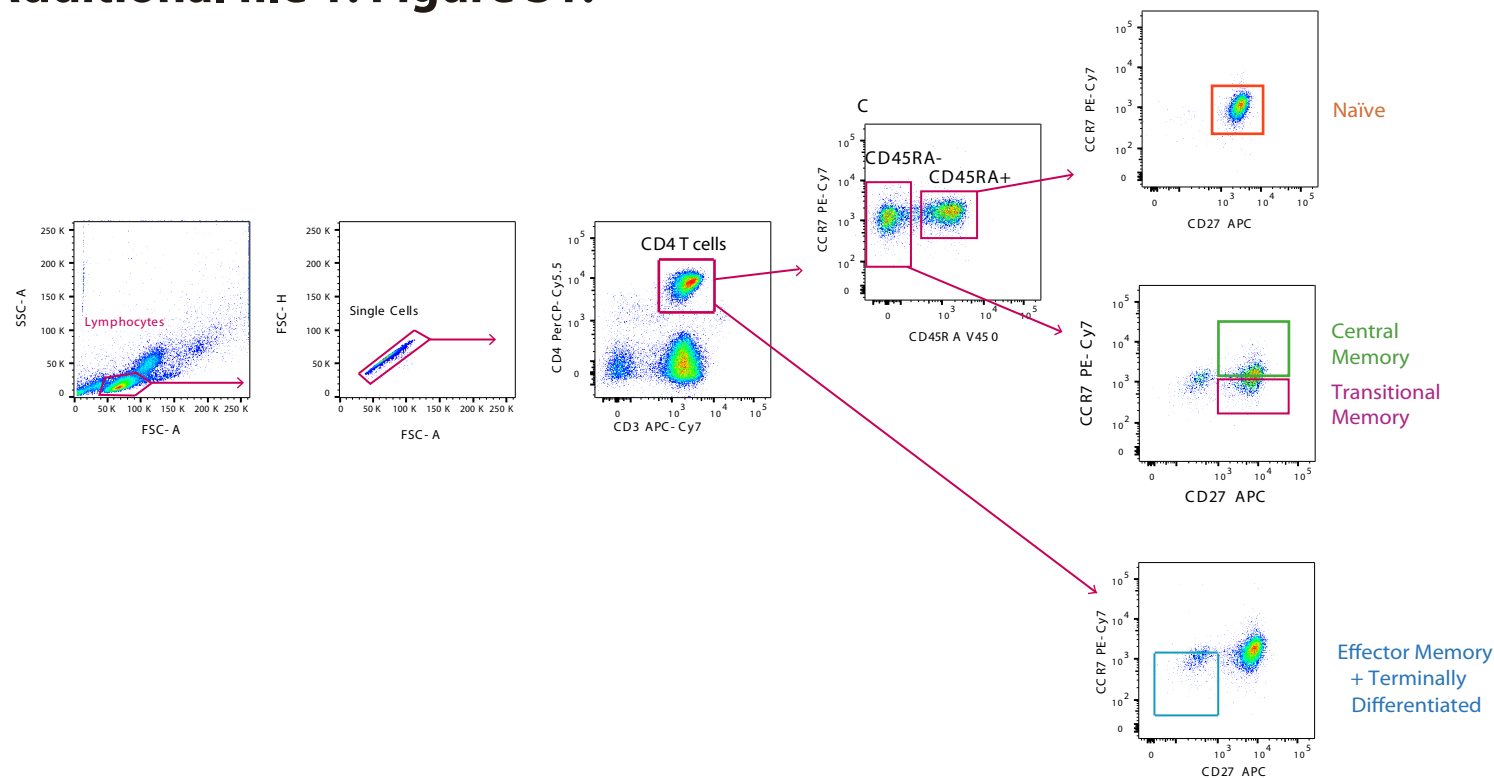

Supplement: Supplementary file 1 — 10.1186/s12977-016-0282-9 Sorting strategy used to purify the different CD4+ T-cell subsets. [file 12977_2016_282_MOESM1_ESM.pdf]

Additional file 2: Figure S2.

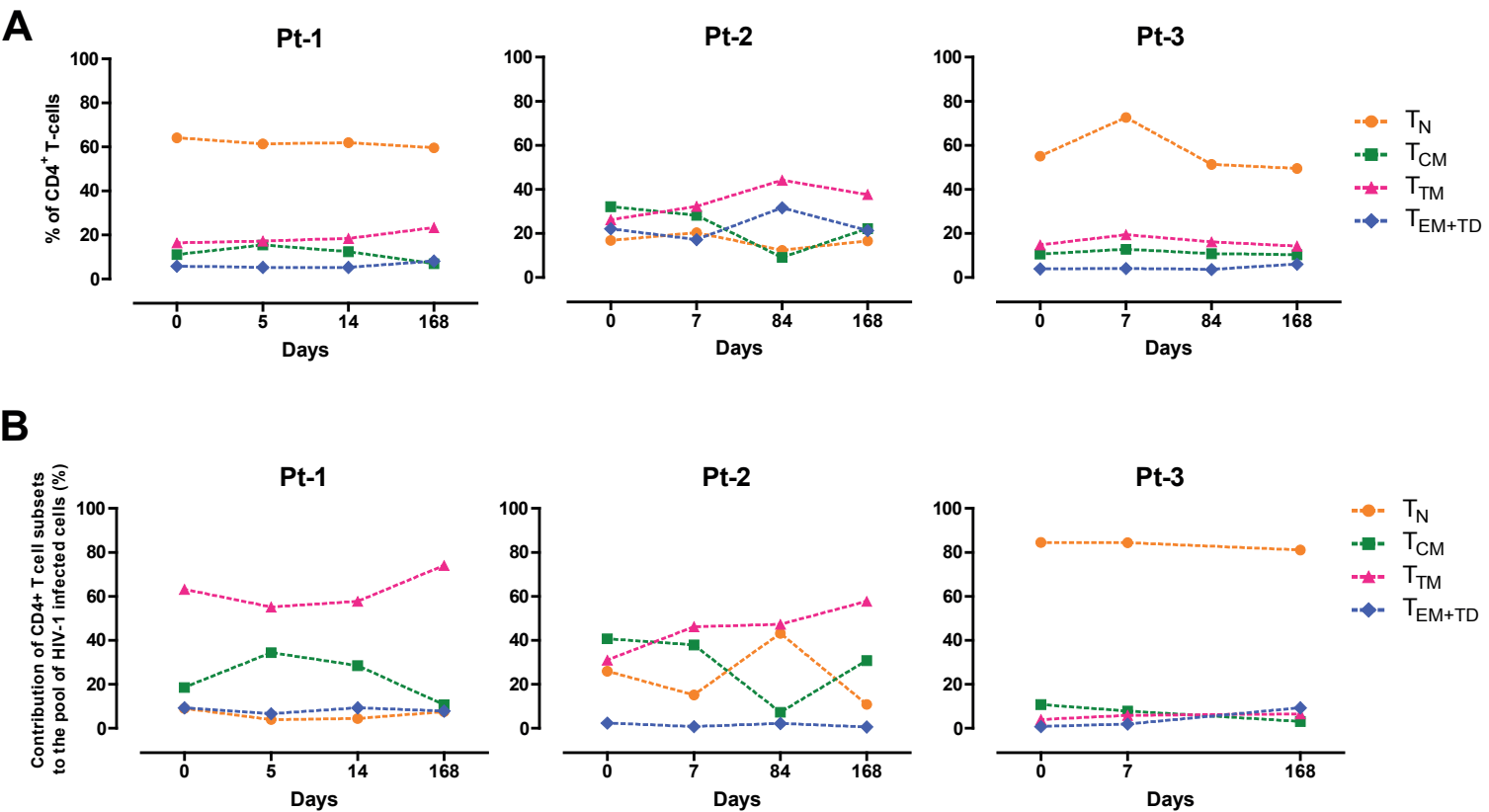

Supplement: Supplementary file 2 — 10.1186/s12977-016-0282-9 Infection dynamics in CD4+ T-cell subsets. A. Frequency of each CD4+ T-cell subset at the different time points after switching treatment, as analyzed by phenotype-based flow cytometry. B. Relative contribution of each CD4+ T-cell subset to the total viral reservoir according to the vDNA content and the frequency of each subset in the whole CD4+ T-cell population at each time point analyzed. [file 12977_2016_282_MOESM2_ESM.pdf]

Additional file 3: Figure S3.

A

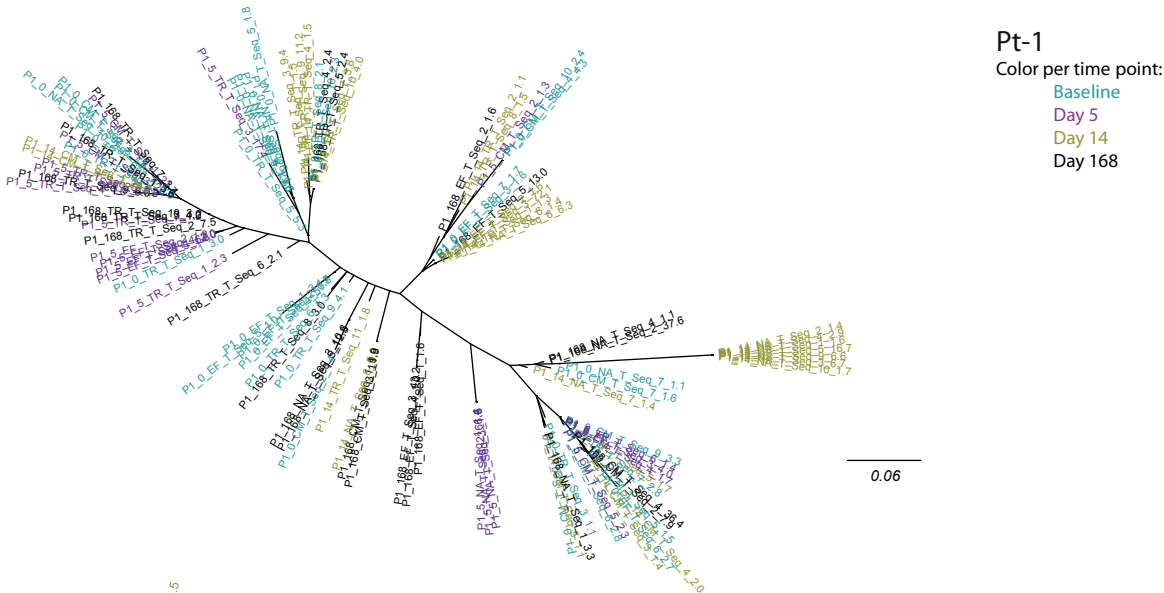

B

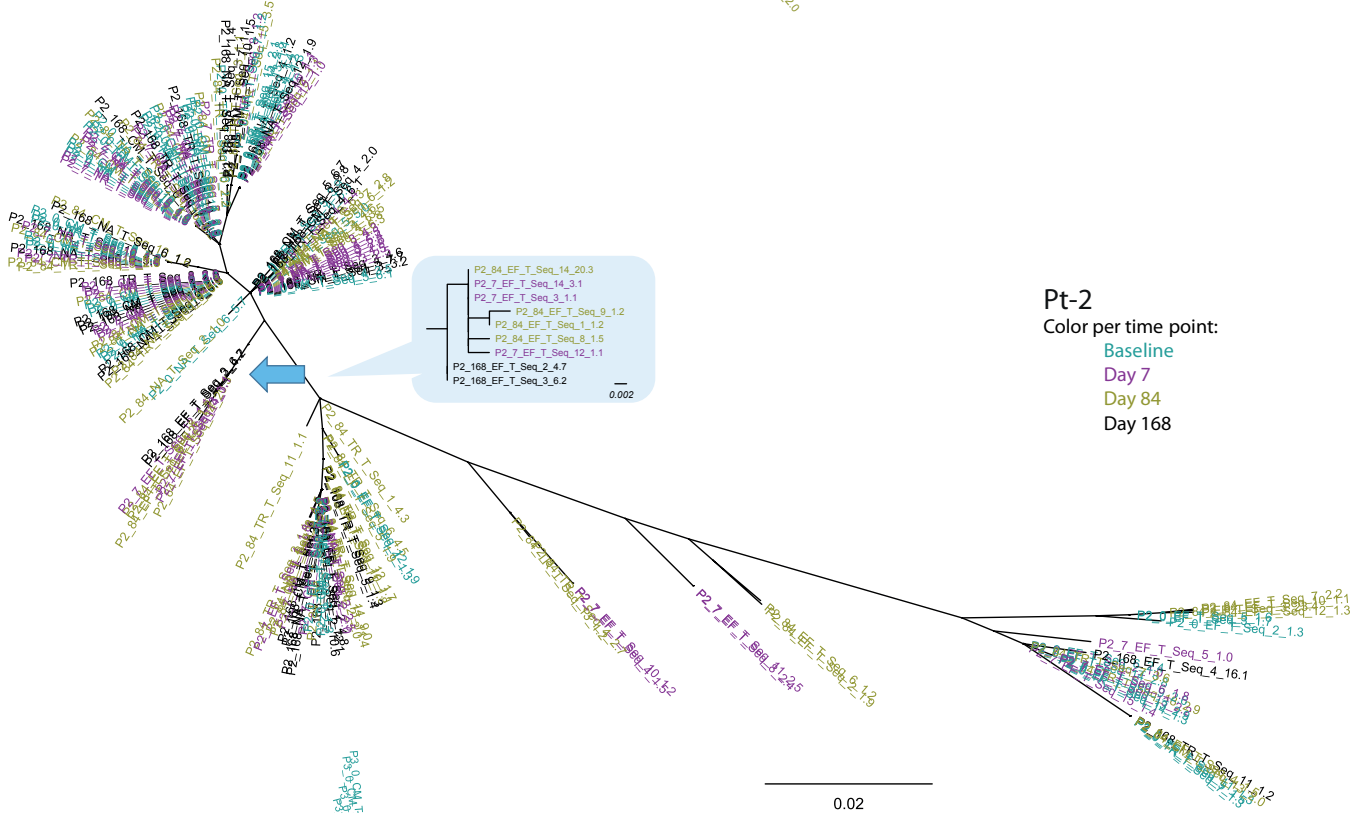

C

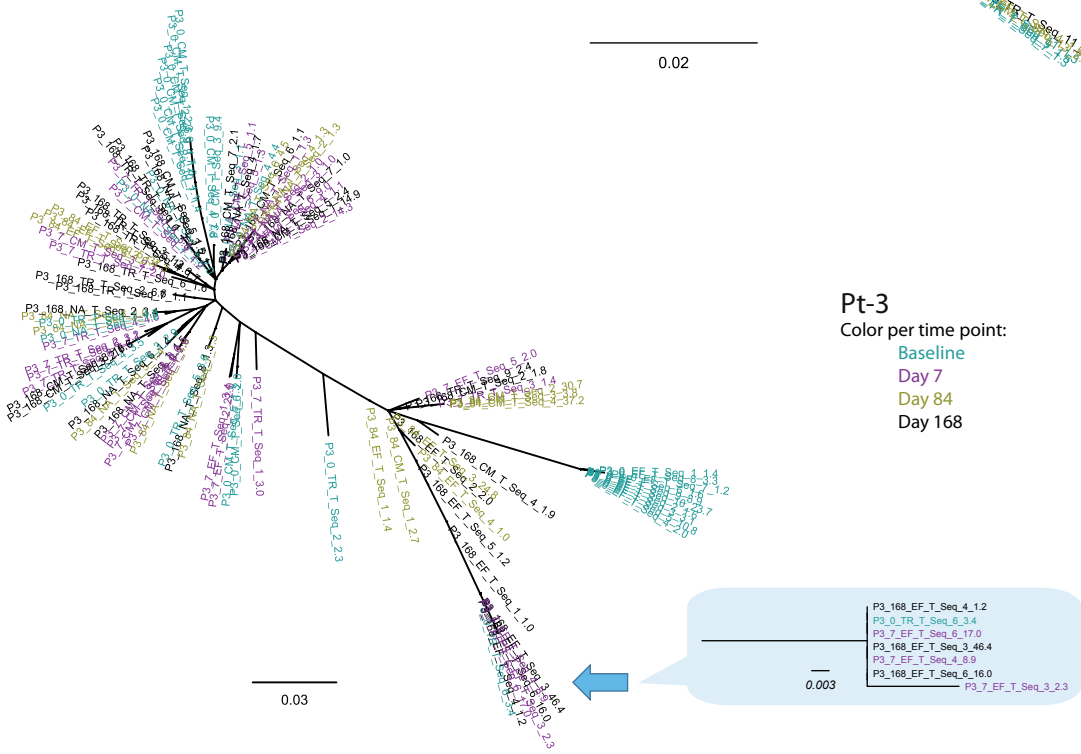

Supplement: Supplementary file 3 — 10.1186/s12977-016-0282-9 Temporal stability of the vDNA integrant pool. In the same phylogenetic tree of Figure 2 the sequences from the four time points analyzed (pre- and post- treatment switching) are identified color-coded. Particular branches, composed mainly by TEM+TD proviral sequences and detected at different time points, are indicated by blue arrows and zoomed in. [file 12977_2016_282_MOESM3_ESM.pdf]

**Additional file 4: Figure S4.**

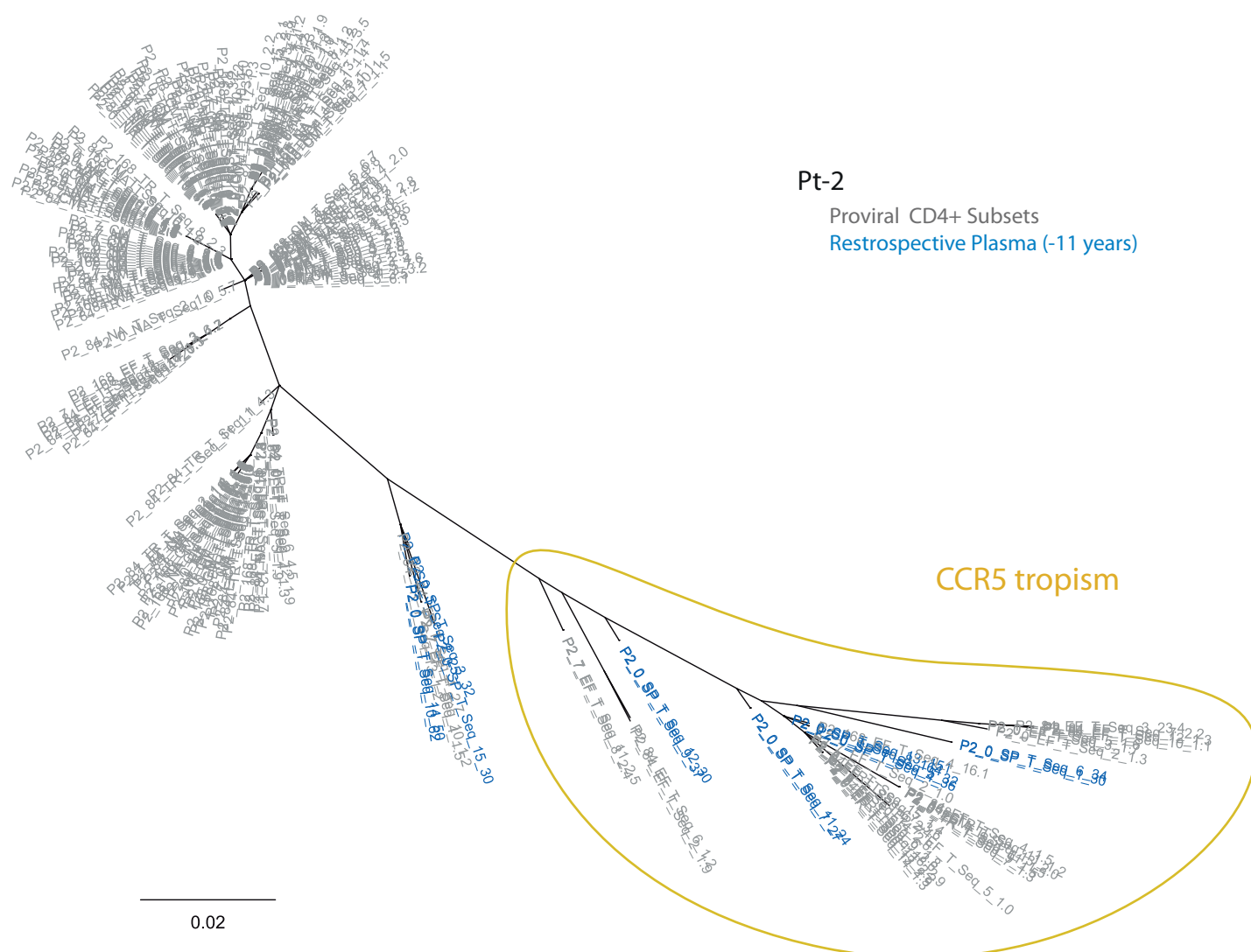

Supplement: Supplementary file 4 — 10.1186/s12977-016-0282-9 Evolution of the co-receptor tropism in Pt-2. Proviral DNA sequences obtained from the four time points have been used to build the maximum likelihood phylogenetic trees used as a backbone. Viral sequences obtained from a retrospective plasma sample are highlighted in blue. The result from the co-receptor tropism prediction is indicated. [file 12977_2016_282_MOESM4_ESM.pdf]
